# Sex and Strain-Specific Variations in Motor Recovery Following Compression Spinal Cord Injury: Comparison of Sprague-Dawley and Wistar Rats

**DOI:** 10.3390/brainsci15020191

**Published:** 2025-02-13

**Authors:** Negin Mojarad, David Doyle, Lucas Gorial Garmo, Ryan Graff, Kayla Reed, Payton Andrew Wolbert, Anusha Uprety, Brynn Stewart, Julien Rossignol, Gary L. Dunbar

**Affiliations:** 1 Field Neurosciences Institute Laboratory for Restorative Neurology, Central Michigan University, Mount Pleasant, MI 48859, USA; mojar1n@cmich.edu (N.M.); doyle2dm@cmich.edu (D.D.); garmo1l@cmich.edu (L.G.G.); graff1rj@cmich.edu (R.G.); reed2kj@cmich.edu (K.R.); wolbe1pa@cmich.edu (P.A.W.); upret1am@cmich.edu (A.U.); stewa3bm@cmich.edu (B.S.); rossi1j@cmich.edu (J.R.); 2 Program in Neuroscience, Central Michigan University, Mount Pleasant, MI 48859, USA; 3 College of Medicine, Central Michigan University, Mount Pleasant, MI 48859, USA; 4 Department of Psychology, Central Michigan University, Mount Pleasant, MI 48859, USA

**Keywords:** compression injury model, spinal cord injury, sex differences, Sprague-Dawley rats, Wistar rats, locomotor activity

## Abstract

**Background/Objectives**: Prior studies have noted varied, spontaneous motor recovery in rat strains after spinal cord injury (SCI), but systematic comparisons of different locomotor measurements across different severity and sexes are lacking. Hence, we quantified hindlimb utilization in male and female Sprague-Dawley (SD) and Wistar rats following moderate and severe SCI. **Methods**: Compression SCI was induced using a 15-g clip for 180 s for moderate SCI or a 50-g aneurysm clip for 60 s for severe SCI in male and female SD and Wistar rats. Measures of locomotor performance using the Basso–Beattie–Bresnahan (BBB), CatWalk gait analysis, and horizontal ladder tests were taken postoperatively and weekly for seven weeks. **Results**: BBB scores indicated greater spontaneous recovery in SD rats, with females showing higher scores than males following moderate and severe SCI. No sex or strain differences were observed in the horizontal ladder test. The CatWalk results indicated greater average hindlimb swing speed in SD rats following moderate SCI, but greater print area was observed in Wistar rats after severe SCI, although female SD rats had greater print area than either male SD or female Wistar rats following moderate SCI. **Conclusions**: The findings that SD rats, especially females, exhibited greater spontaneous motor recovery following moderate SCI indicate the need to consider the sex and strain of rats when conducting therapeutic testing following moderate SCI. The significance of these findings is that they should facilitate the use of appropriate rat models for translational research in SCI that can be applied to future clinical trials.

## 1. Introduction

Spinal cord injury (SCI) leads to temporary or permanent neurological damage, causing sensory, motor, and urinary dysfunctions, as well as sexual impairments [1,2]. According to the 2024 report from the National Spinal Cord Injury Statistical Center, approximately 305,000 individuals currently live with SCI in the United States, with an estimated 18,000 new injuries occurring each year [3]. To date, no definitive treatment has been developed for SCI; existing therapies primarily focus on mitigating the symptoms associated with the injury [4].

Animal models, especially rodent models, are extensively employed for investigating the fundamental pathological changes following SCI and for evaluating the efficacy and safety of therapeutic interventions. While the utilization of animal models allows researchers to focus on specific neuropathological aspects or treatment responses, the translation of therapeutic findings from animal studies to human applications has yielded disappointing outcomes [5]. What is needed is an animal model that accurately mirrors the clinical pathological progression of SCI and adheres to criteria for accessibility and reproducibility [1,6].

Among the SCI models used, mild or moderate injuries often result in relatively short spontaneous recovery times, making it challenging to evaluate the long-term impact of therapeutic interventions. On the other hand, severe injuries can be so extensive that they can prevent any functional recovery, obfuscating the efficacy of promising treatments [7]. Furthermore, rats given mild or moderate SCI often exhibit significant spontaneous recovery relatively soon following the SCI, and this recovery time varies depending on both rat strain and severity of the SCI [8].

Commonly used methods to produce SCIs in rats include complete or partial transections, segment resections, and contusions [9], although the use of compression SCI models has increased recently [10]. The use of aneurysm clip compression provides an efficient way to mimic compression SCIs observed in humans, and the severity of the injury can be easily manipulated by the choosing force of the clip to be used [11]. This method, originally developed for experiments with small animals, enables researchers to precisely control the degree of injury by adjusting the duration of the clip application [12]. A variety of clips with adjustable closing forces are accessible, allowing for the generation of different injury severities in animals [13]. The clip, calibrated for a specific compression force, induces a focal injury and produces significant and sustained deficits as measured by tasks such as the inclined plane test. Studies have shown a strong correlation between functional outcomes, such as scores on the Basso–Beattie–Bresnahan (BBB) test, performance on tasks like the inclined plane and beam walk, and the applied compression force [14].

The aim of this study was to assess whether there were significant sex or strain differences between two commonly used rat models of SCI, specifically comparing male and female SD and Wistar rats following either moderate or severe injury. The study utilized two types of compression clips with two different closing forces to induce varying degrees of SCI severity. The novelty of this study is exemplified in that it represents the first direct comparison of sex differences for spontaneous motor function recovery in BBB, horizontal ladder, and CatWalk test in SD and Wistar rats following moderate and severe SCI.

The present study utilized two different clips, one with a 15-g force given for 180 s and the other with a 50-g force given for 60 s, for inducing a moderate and severe compression SCI, respectively. Both compression SCIs were applied to male and female Wistar and SD rats so that subsequent spontaneous motor performance could be evaluated between the sexes and strains of rats. Established behavioral assessments, such as the BBB test, horizontal ladder walking test, and CatWalk gait analysis, were used to evaluate motor recovery over a 7-week period in an effort to determine which strain and sex might provide the more suitable model for testing potential treatments for compression SCI in rats. The findings of this study are important for facilitating the translation of pre-clinical SCI therapeutic testing to application in human clinical trials, including new developments and prostatic devices [15].

## 2. Materials and Methods

The present study has been designed to compare sex and strain-specific variations in motor function recovery following SCI in SD and Wistar Rats. The study protocol was approved by the Institutional Animal Care and Use Committee (IACUC) at Central Michigan University (Animal Use Protocol #2022-1122).

### 2.1. Subjects

A total of 24 Wistar (Envigo, IN, USA) and SD (Charles River, MA, USA) rats, both male and female, weighing between 200–300 g (approximately 9–12 weeks of age) were utilized. Half of the males and females from each strain were randomly assigned to eight groups of three rats each prior to receiving either moderate or severe compression SCI (Table 1). All rats were housed under standard conditions, including a temperature of 21 °C and a 12 h light/dark cycle, with food and water provided ad lib throughout the 7-week study period. Weekly behavioral assessments were conducted between 1000 and 1400 h during the dark cycle of the rats.

### 2.2. SCI Induction

**Figure 1:**
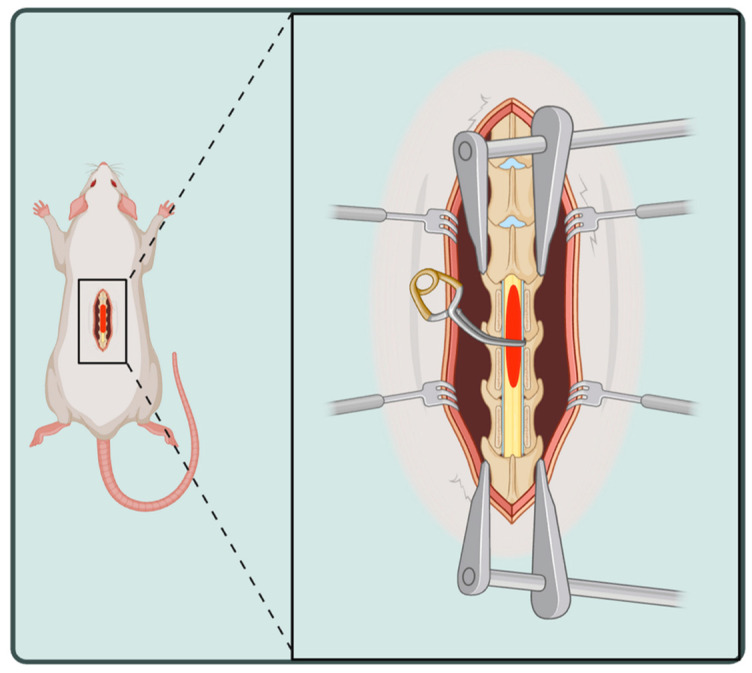
SCI Compression model via clip. Created in BioRender. Mojarradlangroudi, N. (2025) https://BioRender.com/g09e236 (accessed on 10 February 2025).

A mixture of isoflurane 1.5–3% and oxygen (0.8–1 L/min) was administered as anesthesia during surgical compression of the spinal cord. After shaving the back and disinfecting it with alcohol, a laminectomy was then performed at the thoracic 9 level of the spinal cord (Figure 1). To induce a moderate SCI, the vascular clip (F.S.T, 00396-01), with a closing force of 15 g, was applied for 180 s around the spinal cord. In cases of severe injury, the aneurysm clip (Yasargil, FE691K), with a 50-g closing force, was applied for 60 s. Following clip removal, the injury site was washed, the muscles and fascia were sutured using a 4-0 absorbable silk suture, and the incision was closed with sterile 9 mm auto clips. Postoperatively, 0.05 mg/kg of buprenorphine and saline (0.9%; 2 mL) were administered to minimize pain and dehydration, respectively. The animals were then transferred to a recovery cage on top of a warm circulating water pad until they regained consciousness, after which they were moved to a clean cage. All SCI rats received manual bladder massage twice daily until the return of natural bladder function.

### 2.3. Behavioral Evaluations

All behavioral tests were conducted by two experimenters who were blinded to the group identity. Baseline measurements for all tests were given one day before SCI. The baseline consists of a 5 min video recording of animal movement for BBB assessment, three trials on the horizontal ladder test, and three trials in the CatWalk XT^®^ gait system. Behavioral testing started three days after surgery and was given each week throughout the study.

### 2.4. The Basso–Beattie–Bresnahan (BBB) Test

The BBB locomotor rating scale is a well-established 21-point scale designed for assessing motor function in rat models of thoracolumbar SCI [16]. It effectively differentiates phases of recovery based on injury severity and the time elapsed since the injury, making it highly relevant for clinical recovery assessments. The scale is noted for its high sensitivity, reliability in repeated tests, consistency among different raters, and strong validity [17]. In our study, we utilized the BBB test to evaluate motor behavior in rats before surgery and weekly until the end of the seventh week post-surgery. A score of 21 indicates normal motor function, which was the case for all rats at the start of this study. During assessments, a rat was placed in a 90 cm circular plastic container with 20 cm high walls. The movements of each rat were scored independently by two researchers, based on hind limb use, joint movement, paw placement, weight support, and fore/hind limb coordination, using the BBB scale [16]. All videos were recorded for 5 min. The final scores were averaged between the two researchers. Rats that did not show significant motor deficits (i.e., a BBB score above 5) on the first postoperative BBB test were excluded from the study and immediately replaced.

### 2.5. Horizontal Ladder Rung Walking Test

To quantify skilled locomotor movements, rats were trained on the horizontal ladder rung walking test apparatus, a sensitive method for detecting deficits in corticospinal tract connectivity [18]. This test, commonly employed to evaluate locomotor and sensorimotor deficits in adult rats post-SCI, assesses skilled walking, limb placement, and coordination with precision [19]. The horizontal ladder rung walking test apparatus consisted of side walls made of clear Plexiglas 1 m long and 19 cm high when measured from the base of the apparatus to the height of the nine metal rungs, which were each 12 cm long and 3 mm in diameter of and spaced 2 cm from each other [20]. Each rat was given three trials to cross the ladder from a brightly lit starting platform to their dark home cage. The average number of foot slips was used as the dependent measure for this task. Video records were analyzed to confirm the scoring.

### 2.6. CatWalk Gait Analysis

The CatWalk XT^®^ system is an innovative tool for automated gait analysis, offering more detailed parameter specifications compared to earlier behavioral tests [21]. In this experiment, the CatWalk XT^®^ (Noldus Information Technology, Wageningen, The Netherlands) was utilized as a baseline three days before the injury, three days post-surgery, and weekly thereafter until the experiment ended. The system is comprised of a 120 cm (long) × 5 cm (high) horizontal tunnel set on a 130 cm (long) × 20 cm (wide) × 0.5 cm (thick) glass walkway [22]. Animals start at one end and move naturally to the other. The walkway is lit by a green LED, and a high-speed camera beneath it captures the movements of the rat and the prints of their forelimbs (FL) and hindlimbs (HL). The data are stored in a computer and analyzed using CatWalk XT^®^ software (version 10.5; Noldus Information Technology, The Netherlands). In this study, each animal was required to complete at least three uninterrupted walkway crossings at each time point. Detection settings included a maximum run variation of 60%, a camera gain of 20 dB, and a detection threshold of 0.1 arbitrary units (a.u.). Initially, the software automatically recognized footprints, but the manual review was conducted to exclude irrelevant data, such as contacts by the nose, abdomen, or tail. The CatWalk XT^®^ software calculated key parameters related to gait, kinetics, and coordination for each run, with the results averaged for each animal at each time point [23]. Table 2 shows the parameters used in this study. Critical for our study are swing speed and print area, in which increases in either parameter indicate greater recovery [23].

### 2.7. Perfusion and Tissue Processing

For qualitative assessment of the SCIs, rats were euthanized seven weeks after surgery using an overdose of pentobarbital (Fatal Plus, Vortech Pharmaceutical Ltd., Dearborn, MI, USA) and then underwent transcardial perfusion with normal saline and paraformaldehyde containing 4% formalin (Sigma Aldrich Co., St. Louis, MO, USA). Following perfusions, spinal cords were extracted and post-fixed overnight in 4% formalin before being transferred to a 20% and 30% sucrose solution (Thermo Fisher Co., Ward Hill, MA, USA). After equilibration in the sucrose solution, the spinal cords were cut into 2 cm longitudinal sections and frozen in methyl butane (Sigma Aldrich Co., St. Louis, MO, USA), then stored at −80 °C. The spinal cords were embedded in Tissue-Tek^®^ O.C.T. Compound Embedding medium (Sakura Finetek USA, Inc., Torrance, CA, USA) and cut into 30 μm transverse sections. Hematoxylin and Eosin staining [24] was performed to ensure comparable lesion placement. Representative microphotographs from the H&E sections were taken to qualitatively compare moderate and severe lesions in males and females in both SD and Wistar rat samples.

### 2.8. Statistical Analysis

Data were analyzed using IBM SPSS Statistics (Version 29). A three-way analysis of variance (ANOVA) was conducted to evaluate the interactive effects of time, strain, and sex on various outcome measures, including pre-surgery and weekly averages up to the 7th week. Post hoc comparisons using the least significant difference (LSD) were performed to assess significant main effects and interactions identified in the analyses. A repeated-measures design was utilized to compare group effects across different time points. Profile plots with error bars visualized mean changes over time by group. A significance level of *p* < 0.05 was established for all analyses. For any analysis revealing a significance of *p* < 0.05 on Mauchly’s test of sphericity, the Greenhouse–Geisser correction was applied, and the adjusted degrees of freedom were reported.

## 3. Results

### 3.1. Moderate Injury

Four rats (one male SD and two male and one female Wistar) were excluded from all analyses because they had insufficient deficits (a BBB score greater than 5) during the initial postoperative BBB testing. These were immediately replaced with rats in each of these categories that had sufficient deficits (scores of 5 or less on the BBB test).

#### 3.1.1. BBB Test Results in Moderate Injury Groups

**Figure 2:**
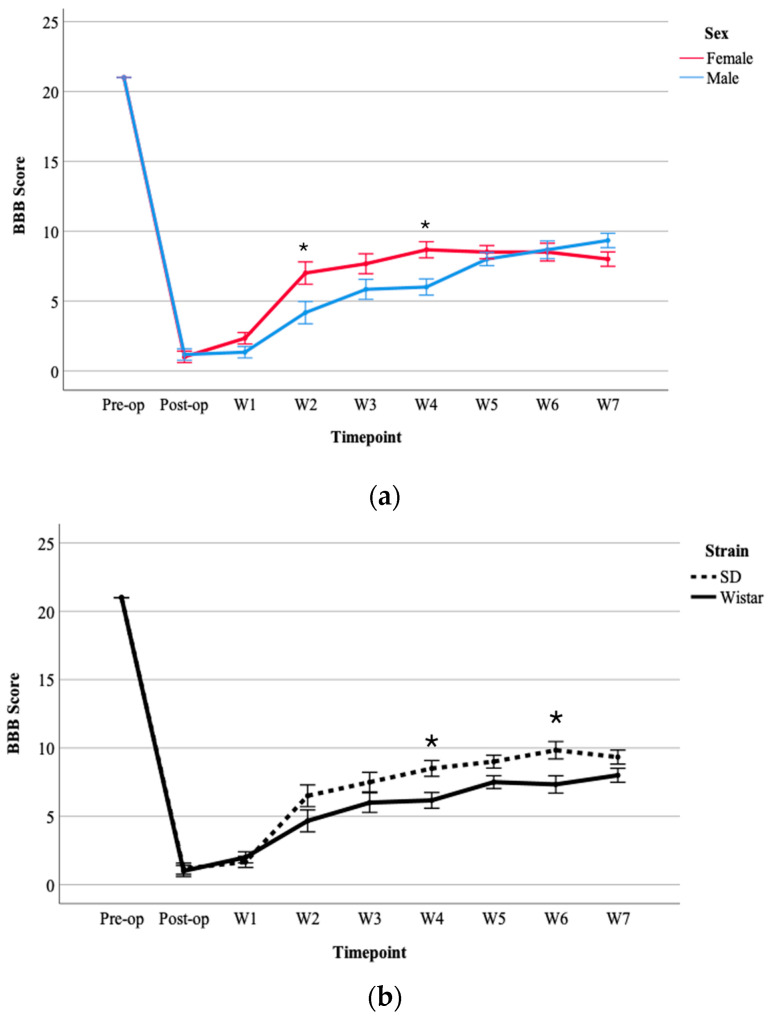
(**a**) Comparison between males and females following moderate SCI in 7 weeks; (**b**) comparison between SD and Wistar rats following moderate SCI. Values = M ± SEM. * shows significant differences within the groups in *p* < 0.05 level.

A three-way repeated measures ANOVA was performed to determine the effects of time, sex, and strain on BBB scores following moderate SCI. Although the ANOVA revealed no significant three-way interaction between time, sex, and strain, *F*(8, 64) = 0.468, *p* = 0.874, statistically significant two-way interactions were found between time and sex, *F*(8, 64) = 5.331, *p* < 0.001 and with significant differences between the two sexes observed at week 2 (*p* = 0.037) and week 4 (*p* = 0.011) (Figure 2a). Additionally, there was a significant interaction between time and strain, *F*(8, 64) = 2.831, *p* = 0.009 (Figure 2b). Three days post-moderate-SCI, both strains exhibited a significant decrease in motor scores (BBB) compared to their pre-injury scores. The results indicated significant differences between the two strains at week 4 (*p* = 0.021) and week 6 (*p* = 0.024). SD rats demonstrated greater spontaneous motor function recovery compared to Wistar rats (Figure 2b).

#### 3.1.2. Horizontal Ladder Test Results in Moderate Injury Group

A three-way repeated measures ANOVA was performed to determine the effects of time, sex, and strain on horizontal ladder performance following moderate SCI. The ANOVA revealed no significant three-way interaction between time, sex, and strain [*F*(8, 64) = 0.871, *p* = 0.546]. Moreover, no statistically significant two-way interactions were found between time and sex [*F*(8, 64) = 1.136, *p* = 0.352] nor between time and strain [*F*(8, 64) = 1.463, *p* = 0.188] (Table 3).

#### 3.1.3. Catwalk Test Results

Hindlimb swing speed in the moderate injury group

**Figure 3:**
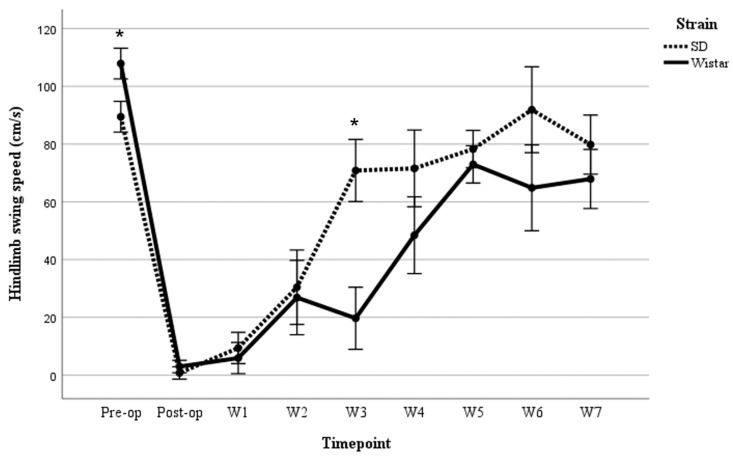
Comparison between males and females in Wistar rats in swing speed following moderate SCI in 7 weeks. Values = M ± SEM. * Shows significant differences within the groups in *p* < 0.05 level.

A three-way repeated measures ANOVA was performed to determine the effects of time, sex, and strain on hindlimb swing speed following moderate SCI. Mauchly’s test of sphericity indicated that the assumption of sphericity was not met [*χ*^2^(35) = 79.289, *p* < 0.001]. Thus, degrees of freedom were corrected using the Greenhouse–Geisser estimate of sphericity (*ε* = 0.436). The corrected ANOVA revealed no significant three-way interaction between time, sex, and strain [*F*(3.489, 27.909) = 2.636, *p* = 0.062], partial *η*^2^ = 0.248. No statistically significant two-way interaction was found between time and sex [*F*(3.489, 27.909) = 1.538, *p* = 0.223], partial *η*^2^ = 0.161; however, a statistically significant two-way interaction between time and strain was demonstrated [*F*(3.489, 27.909) = 3.063, *p* = 0.038], partial *η*^2^ = 0.277. Post hoc analyses indicated that SD rats demonstrated significantly slower hindlimb swing speed than Wistar rats on pre-surgery assessment, *p* = 0.040, while the average hindlimb swing speed of SD rats was significantly greater than that of Wistar rats on post-SCI week 3 (*p* = 0.010) (Figure 3).

Hindlimb print area in moderate injury

**Figure 4:**
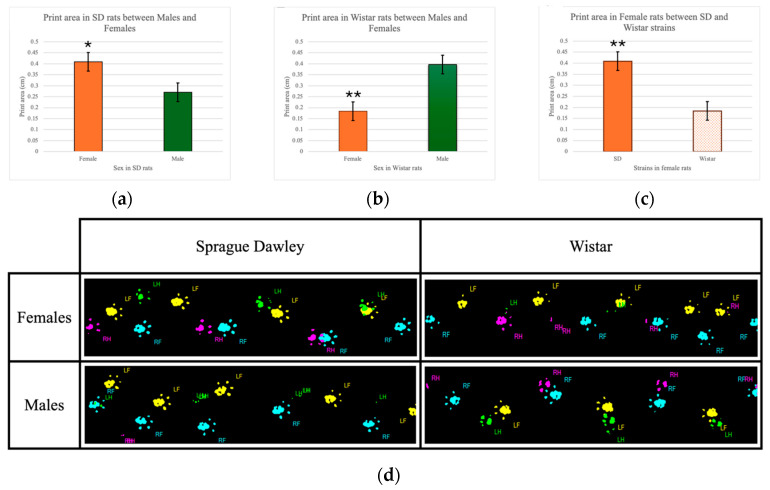
(**a**) Print area comparison between male and female in SD rats following moderate SCI; (**b**) Print area comparison between male and female in Wistar rats following moderate SCI; (**c**) Print area comparison between SD and Wistar rats in females following moderate SCI. Values = M ± SEM. * Shows significant differences within the groups in *p* < 0.05 level. ** Shows significant differences within the groups in *p* < 0.01 level. (**d**) SD and Wistar rats print area. RF: Right Front limb (Blue color), LF: Left Front limb (Yellow color), RH: Right Hind limb (Pink color), LH: Right Hind limb (Green color).

A three-way repeated measures ANOVA was performed to determine the effects of time, sex, and strain on hindlimb print length following moderate SCI. Mauchly’s test of sphericity indicated that the assumption of sphericity was not met [*χ*^2^(35) = 83.502, *p* < 0.001]. Thus, degrees of freedom were corrected using the Greenhouse–Geisser estimate of sphericity (*ε* = 0.228). The corrected ANOVA revealed no significant three-way interaction between time, sex, and strain [*F*(1.823, 14.581) = 0.519, *p* = 0.589], partial *η*^2^ = 0.061. No statistically significant interaction was found between time and sex [*F*(1.823, 14.581) = 0.531, *p* = 0.583], partial *η*^2^ = 0.062. However, a statistically significant interaction between subjects, sex, and strain was observed. Irrespective of time, female SD rats (M = 0.409; SE = 0.042) showed 0.139 cm^2^ larger hindlimb print areas than male SD rats (M = 0.270; SE = 0.042), *p* = 0.047 (Figure 4a). Conversely, female Wistar rats (M = 0.184; SE = 0.042) showed 0.213 cm^2^ smaller hindlimb print areas than male Wistar rats (M = 0.397; SE = 0.042), *p* = 0.007 (Figure 4b). Between strains, female SD rats demonstrated 0.225 cm^2^ larger hindlimb print areas than female Wistar rats (*p* = 0.005). No significant difference in hindlimb print area was seen between strains in male rats (*p* = 0.064) (Figure 4c). Figure 4d shows the right and left paw prints in male and female rats in both strains.

Other CatWalk gait parameters in moderate injury

In addition to the significant findings related to swing speed and hindlimb print area following moderate injury, our analysis revealed notable differences across several other gait parameters. These parameters, including run duration, run average speed, run maximum variation, hindlimb print length, and hindlimb print width, demonstrated significant interactions between time, sex, and strain across various parameters. Table 4 summarizes these key results.

#### 3.1.4. Lesion Site Staining Results in Moderate Groups

**Figure 5:**
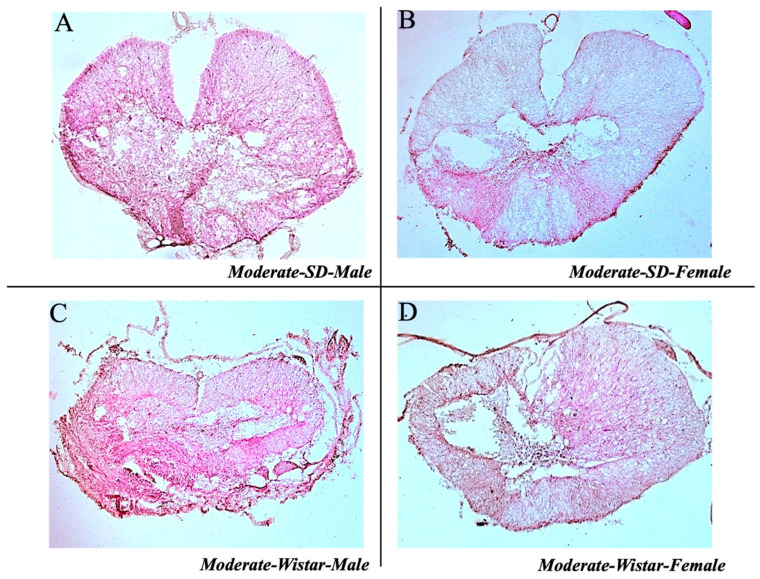
H&E staining in transverse sections of Sprague Dawley and Wistar rats in Moderate SCI. (**A**) Shows the injury site of SD male rats in moderate injury. (**B**) Shows the injury site of SD female rats in moderate injury. (**C**) Shows the injury site of Wistar male rats in moderate injury. (**D**) Shows the injury site of Wistar female rats in moderate injury.

The images depicting transverse sections of the lesion via H&E staining reveal the site epicenter for Wistar and SD male and female rats in the moderate injury group (Figure 5) and provide a qualitative assessment of consistent injury severity at the surgical site. By visualizing the tissue architecture and the extent of damage, the efficacy of the surgical procedures was confirmed.

### 3.2. Severe Injury

Three SD rats died within 20–30 days after severe surgery and were immediately replaced.

#### 3.2.1. BBB Test Results in Severe Injury Group

**Figure 6:**
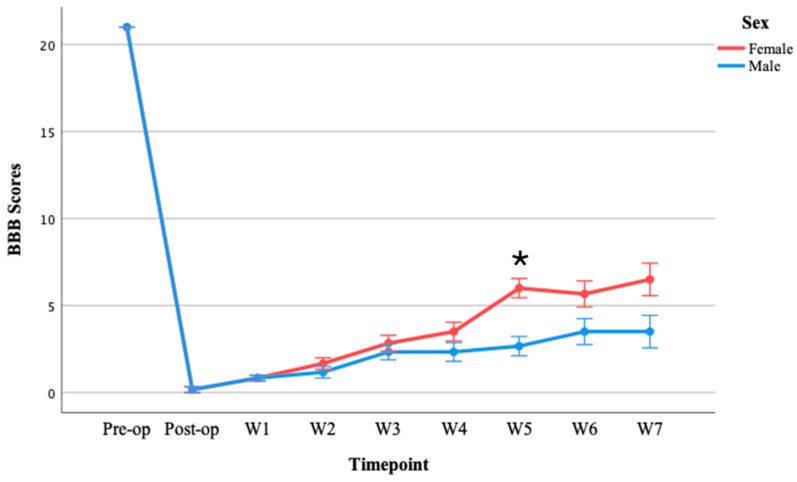
Comparison of BBB scores between males and females following severe SCI in 7 weeks. Values = M ± SEM. * Shows significant differences within the groups in *p* < 0.05 level.

A three-way repeated measures ANOVA was performed to determine the effects of time, sex, and strain on BBB scores following severe SCI. Mauchly’s test of sphericity indicated that the assumption of sphericity was not met [*χ*^2^(35) = 60.068, *p* = 0.019]. Thus, degrees of freedom were corrected using the Greenhouse–Geisser estimate of sphericity (*ε* = 0.358). The corrected ANOVA revealed no significant three-way interaction between time, sex, and strain [*F*(2.867, 22.936) = 2.783, *p* = 0.066], partial *η*^2^ = 0.258. No statistically significant two-way interaction was found between time and strain [*F*(2.867, 22.936) = 0.506, *p* = 0.674], partial *η*^2^ = 0.059. However, a statistically significant two-way interaction between time and sex was demonstrated [*F*(2.867, 22.936) = 4.255, *p* = 0.017], partial *η*^2^ = 0.347. Female rats demonstrated significantly higher BBB scores than males on post-injury week 5 (*p* = 0.003) (Figure 6).

#### 3.2.2. Horizontal Ladder Test Results in Severe Injury Group

A three-way repeated measures ANOVA was performed to determine the effects of time, sex, and strain on horizontal ladder performance following Severe SCI. The ANOVA revealed no significant three-way interaction between time, sex, and strain [*F*(8, 64) = 1.838, *p* = 0.086]. Moreover, no statistically significant two-way interactions were found between time and sex [*F*(8, 64) = 1.161, *p* = 0.336] nor between time and strain [*F*(8, 64) = 0.914, *p* = 0.511] (Table 5).

#### 3.2.3. Catwalk Test Results

Hindlimb swing speed in severe injury

**Figure 7:**
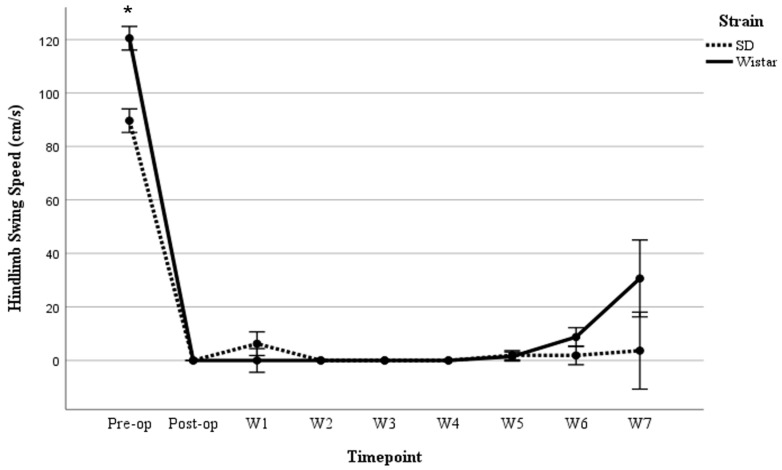
Comparison of swing speed of catwalk test between SD and Wistar rats following severe SCI in 7 weeks. Values = M ± SEM. * *p* < 0.05.

A three-way repeated measures ANOVA was performed to determine the effects of time, sex, and strain on hindlimb swing speed following severe SCI. The ANOVA revealed no significant three-way interaction between time, sex, and strain [*F*(8, 64) = 0.627, *p* = 0.752]. No statistically significant two-way interaction was found between time and sex [*F*(8, 64) = 0.240, *p* = 0.982]. However, a statistically significant two-way interaction between time and strain was observed [*F*(8, 64) = 2.677, *p* = 0.013], revealing that Wistar rats had higher pre-SCI swing speed scores than Wistar rats (*p* = 0.001). However, no significant differences in swing speed scores were seen between the two strains at any time point following severe SCI (Figure 7).

Hindlimb print area in severe injury

**Figure 8:**
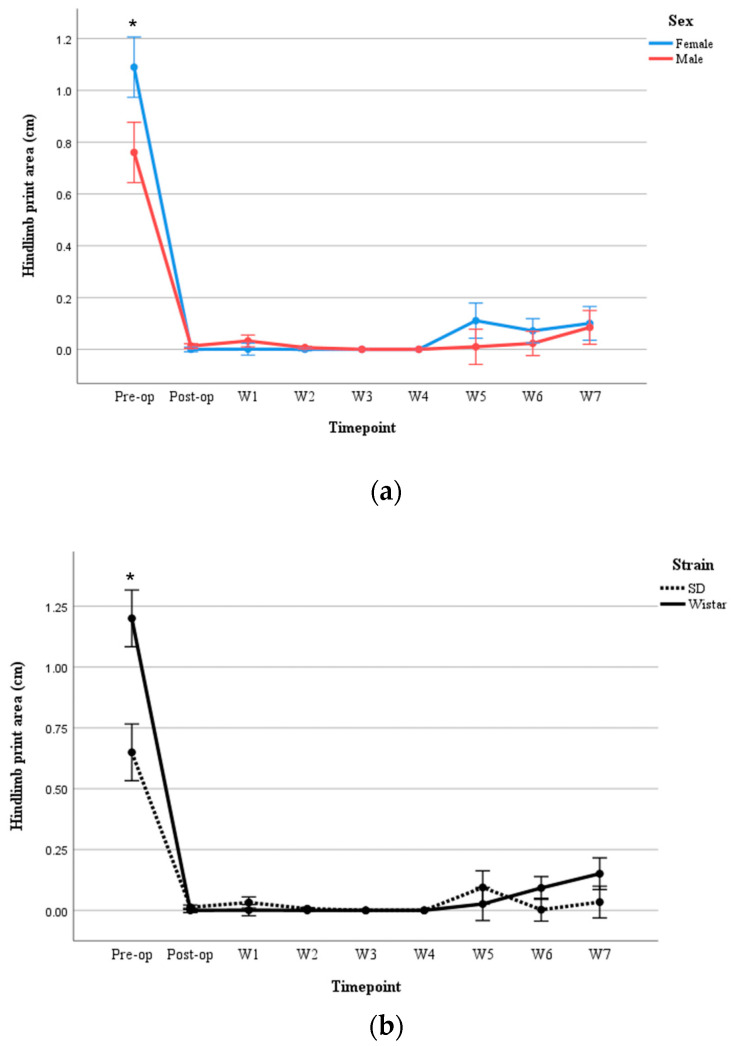
(**a**) Comparison of hindlimb print area between males and females following severe SCI; (**b**) comparison between SD and Wistar rats following severe SCI; values = M ± SEM; * *p* < 0.05.

A three-way repeated measures ANOVA revealed no significant interaction between time, sex, and strain [*F*(8, 64) = 0.795, *p* = 0.609]. However, significant two-way interactions were found between time and sex [*F*(8, 64) = 2.336, *p* = 0.029] (Figure 8a) and between time and strain [*F*(8, 64) = 6.628, *p* < 0.001] (Figure 8b) for baseline performance, with Wistar rats having 0.551 cm^2^ larger hindlimb paw areas than SD (*p* = 0.010). However, no significant differences were seen at any post-injury time point. 

Other CatWalk gait parameters in severe injury

Table 6 summarizes significant findings from the CatWalk gait analysis, highlighting the interactions between time, sex, and strain across various parameters between SD and Wistar rats, as well as between male and female rats, across different time points following severe SCI. These parameters include step sequence, average max contact area, and average step cycle.

#### 3.2.4. Lesion Site Staining Results in Severe Injury

**Figure 9:**
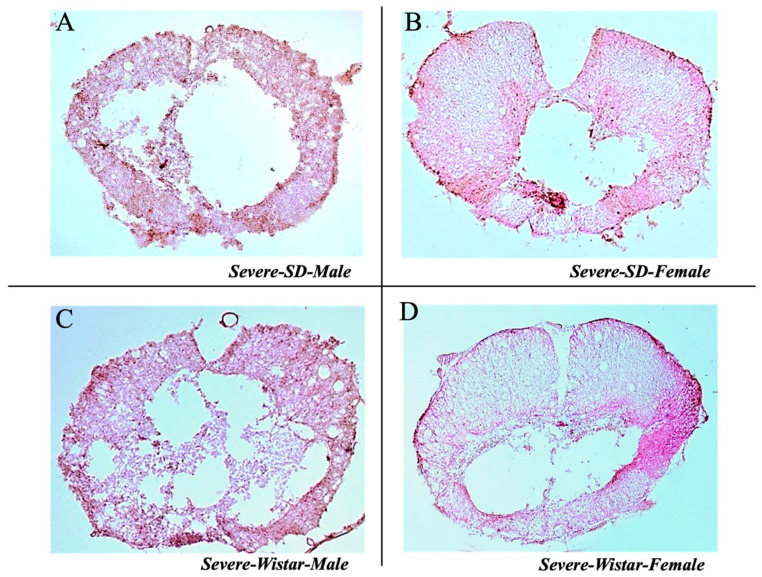
H&E staining in transverse sections of Sprague-Dawley and Wistar rats in severe SCI. (**A**) Injury site of SD male rats in severe injury. (**B**) Injury site of SD female rats in severe injury. (**C**) Injury site of Wistar male rats in moderate injury. (**D**) Injury site of Wistar female rats in severe injury.

Figure 9 displays images of transverse sections from the epicenter of the lesion site for male and female Wistar and SD rats in the severe injury group, following H&E staining, indicating that injury severity at the surgical site was similar for each group of rats. In addition, the figure indicates that the severe lesions were noticeably more extensive than the moderate lesions.

## 4. Discussion

The findings of the present study demonstrate that the varying clip compressions with different closing forces induce different severity levels of SCI in rats, confirming the degree of control provided by using an aneurysm clip with optimal force and compression time that can mimic mild, moderate, or severe SCI. In our study, the 15-g force for 180 s provided a moderate SCI, producing significant deficits with a modest degree of spontaneous recovery, while the 50-g force clip for 60 s induced a severe SCI with little evidence of spontaneous recovery. Notably, SD rats exhibited greater spontaneous motor recovery following moderate SCI compared to Wistar rats, with significant differences observed in both BBB recovery scores and gait parameters measured on the Catwalk test. Furthermore, the scores for females indicated greater recovery than males on most measures following moderate SCI.

These results align with previous findings showing that the clip compression model allows for a high degree of control over injury severity by adjusting the closing force and duration of compression [12,25]. The ability to precisely modulate the degree of compression offers significant advantages for studying the mechanisms of recovery and assessing the efficacy of potential therapeutic interventions in a pre-clinical setting. Additionally, the use of aneurysm clip compression closely mimics compression SCIs in humans [7]. Our results support the contention that this model remains a highly reliable method for studying SCI and exploring novel therapeutic approaches.

Based on our results, it has become apparent that the degree of spontaneous motor recovery is related to the strain and sex of the rat following moderate compression SCI. In the current study, SD rats exhibited greater spontaneous motor recovery in the BBB test and higher swing speed, body speed, and print paw metrics in the catwalk test compared to Wistar rats following moderate SCI. Our results support the findings of Mills and colleagues [8], who observed faster and more complete recovery of BBB scores in SD rats than in Wistar or Long-Evans rats following T10 contusion and T13 hemisection SCIs. These authors suggested that certain rat strains may possess cell populations or subpopulations that are more prone to injury, resulting in different behavioral outcomes. Similarly, Mestre and colleagues [26] found that the BBB scores of SD rats indicated faster recovery relative to Lewis or Fisher 344 rats following T9 contusions.

In agreement with previous studies reporting faster recovery in SD rats following contusion injury to the spinal cord, our results indicated that SD rats also recovered faster in a compression model of SCI. Furthermore, our study demonstrated that female SD rats exhibited significantly larger hindlimb print areas compared to both male SD rats and female Wistar rats following moderate compression SCI. Previous work has indicated that female Fisher rats had greater tissue preservation, including spared ventral white matter at the lesion center, which correlated with higher BBB and CatWalk hindlimb scores following T8 contusions [27].

Although severe SCI typically results in significantly limited functional improvements [7], our research revealed that female rats exhibited notably higher BBB scores compared to males, with no significant strain differences observed following severe compression injury. This underscores the importance of noting that sex differences can affect the level of spontaneous recovery following severe spinal cord compression, which should be considered when evaluating potential therapeutic interventions following SCI. Zheng and colleagues [23] explored CatWalk parameters in female Wistar rats subjected to severe cervical compression SCI using a 28-g clip. They found strong correlations between CatWalk parameters, particularly swing speed, and BBB scores with tissue preservation [23]. Conversely, their 2023 study on thoracic SCI indicated that CatWalk parameters, including regularity index, stride length, base of support, and swing speed, demonstrated only moderate to poor correlations with BBB scores. They posited that the severity of thoracic SCI, characterized by persistently low BBB scores (<8), may diminish the sensitivity of the BBB scale to detect subtle improvements in coordination and overall locomotor recovery. These authors argued that static print parameters, such as print intensity and size, could offer a more reliable means of assessing functional recovery in cases of severe thoracic compression or contusion SCI [28].

The reason that SD rats demonstrate more spontaneous motor function recovery compared to Wistar rats is currently unknown. One hypothesis is that SD rats exhibit a more substantial increase in the expression of genes related to myelin structural proteins following SCI, accelerating neural repair and regeneration relative to other rat strains [29]. For example, Lewis rats show no such change in gene expression following SCI, suggesting the unique regenerative capacity of SD rats that likely contributes to their improved recovery in BBB assessments [29].

Although we did observe that Wistar rats had larger paw-print areas than SD rats during baseline training in the CatWalk task, this did not mitigate the greater deficits that were later observed in the Wistar rats following SCI. Nonetheless, this finding does suggest that inherent strain differences may affect subsequent recovery in rats with SCIs. Interestingly, Koopmans and colleagues [30] also observed strain differences in locomotor performance on the CatWalk tasks in rats without injury. These investigators found that Wistar rats had shorter hindlimb swing phase durations on the CatWalk test compared with Lewis and Wistar rats. These findings underscore the importance of providing baseline testing, as inherent strain differences could interfere with the recovery process following SCI [30]. Our findings that females had increased paw-print area during baseline testing in the CatWalk task also underscore that both inherent sex and strain differences need to be factored into gauging the spontaneous recovery following SCI.

Although it is tempting to interpret our findings of more spontaneous recovery in female rats as hormonal differences between the sexes, more evidence is needed to support that hypothesis. In support of the hypothesis that female hormones can accelerate recovery after SCI, Chaovipoch and colleagues [27] gave two-month-old and one-year-old female rats an ovariectomy and implanted with silastic capsules containing 17β-estradiol or a vehicle, followed by a complete crush injury at T8–9. The administration of 17β-estradiol improved hindlimb locomotor recovery, increased white matter sparing, and decreased apoptosis in both pre- and post-menopausal rats, which highlights the role of hormones in motor function recovery. Nonetheless, it is important to consider that the observed gender differences following SCI may not be entirely attributed to female sex hormones, or their influence, at least, might be limited [27]. Contrary to these findings, Swartz and co-workers [31] observed no effect of chronic circulating levels of estrogen on BBB scores or morphological measures following T10 SCI in either male or female rats. Clearly, the role of sex (as well as strain) differences will require further study in order to fully elucidate their underlying roles in recovery after SCI.

The applications of these findings should facilitate more appropriate uses of sex and strain of rats for pre-clinical therapeutic assessments, particularly those that test the long-term efficacy of potential treatments following SCI. This should culminate in a more reliable translation of findings from rat models of SCI to clinical applications in human trials.

A major strength of this study is that it demonstrated significant differences in spontaneous motor recovery in two strains of rats (SD and Wistar) following two levels of severity following compression injury of the spinal cord. In general, Wistar rats, particularly female rats, showed significantly greater spontaneous recovery than SD rats. However, future studies are needed to address the limitations of the study. First, it would be beneficial to include additional rat strains and both sexes for a broader comparison. Secondly, incorporating tissue evaluations to assess pathophysiology, inflammation, glial scarring, and cavity formation would provide valuable insights into the underlying mechanisms of recovery. Studies in our lab are being designed to utilize the findings of the present study for application in upcoming pre-clinical therapeutics experiments for various treatments following SCI. Specifically, we will be testing whether laser therapy, stem cell treatment, and functional electrical stimulation can facilitate recovery following SCI in male and female Wistar rats. We expect the results of these experiments to provide a more reliable assessment of the therapeutic efficacy, which should increase the success of its translation to clinical trials.

## 5. Conclusions

The findings that the clip compression model is a valuable tool for precisely modulating injury severity, our study indicated that SD rats exhibited greater spontaneous motor recovery than Wistar rats following moderate SCI, with significant differences in recovery scores and gait parameters. Additionally, females consistently outperformed males in recovery assessments across injury groups, highlighting the influence of sex on motor recovery. In conclusion, our findings underscore the importance of sex, strain-specific responses, and injury severity in SCI research., enabling detailed investigations into recovery mechanisms and therapeutic interventions, ultimately enhancing the translational potential of pre-clinical SCI studies to human clinical trials.

## Figures and Tables

**Table 1:** Study groups.

Injury Severity	Strain	Sex
Moderate	Wistar	Males (n = 3) Females (n = 3)
	Sprague-Dawley	Males (n = 3) Females (n = 3)
Severe	Wistar	Males (n = 3) Females (n = 3)
	Sprague-Dawley	Males (n = 3) Females (n = 3)

**Table 2:** CatWalk parameters and their descriptions.

Parameter	Description
Swing speed (cm/s)	The velocity of the paw during the swing phase of the gait cycle.
Print area (cm)	The surface area of the paw print.
Print length (cm)	The rostral-caudal measurement of the paw print.
Print width (cm)	The lateral-medial measurement of the paw print.
Run Duration (s)	The total amount of time it takes for the animal to complete a single walk across the walkway.
Run Average Speed (cm/s)	The mean velocity at which the animal progresses throughout the entire duration of the run.
Run Max Variation (cm/s)	The maximum variation observed in speed or gait pattern throughout the run.
Maximum contact area (cm^2^)	The largest area of the hindlimb paw in contact with the walkway during a step.
Body speed (cm/s)	Calculated by dividing the distance that the animal’s body traveled from one initial contact of a paw to the next by the time to travel that distance.
Duty cycle (%)	Expresses the stand as a percentage of the step cycle.
Step cycle (s)	The total time for one complete movement cycle from the initial contact of one paw to the next contact of the same paw.

**Table 3:** Mean comparison between males and females in SD and Wistar strains following moderate SCI.

Groups	Males (Mean, % Error)	Females (Mean, % Error)	Δ Mean (Male–Female) [95% CI]	*p*-Value
SD	56.78	44.21	12.57 [−17.00–42.13]	0.356
Wistar	45.36	59.55	−14.19 [−43.75–15.38]	0.301
Δ Mean (SD–Wistar) [95% CI]	11.41 [−18.15–40.97]	−15.34 [−44.90–14.22]	---	*---*
*p*-value	0.399	0.266	*---*	*---*

**Table 4:** Significant interactions for additional CatWalk gait parameters assessed in male and female rats in both strains SD and Wistar following moderate SCI in 7 weeks.

Parameter	Interaction/Effect	Key Observations
Run Duration (s)	Time*Sex	Males had longer run durations on week 4 (*p* = 0.032), week 5 (*p* = 0.016), week 6 (*p* = 0.008), and week 7 (*p* = 0.034).
Run Average speed (cm/s)	Time*Sex	Males had slower run speeds on week 4 (*p* = 0.011), week 5 (*p* < 0.001), week 6 (*p* = 0.004), and week 7 (*p* = 0.045).
	Sex	Males (*M* = 17.842; *SE* = 1.814) ran an average of 6.286 cm/s slower than females (*M* = 24.128; *SE* = 1.814).
Run Max Variation (cm/s)	Time*Sex*Strain	Female SD rats were significantly higher than male SD rats at week 2 (*p* = 0.020). Female Wistar rats were significantly lower than male Wistar rats at week 2 (*p* = 0.041). Female Wistar rats had significantly greater maximum variation in run speed than female SD rats at week 7 (*p* = 0.017), Male Wistar rats had significantly greater maximum variation in run speed than male SD rats at week 2 (*p* = 0.008).
Hindlimb print length (cm)	Time*Strain	On post-SCI week 4, SD rats demonstrated significantly greater hindlimb print lengths than Wistar rats, *p* = 0.040.
	Sex*Strain	Female SD rats (M = 1.150; SE = 0.152) showed 0.528 cm longer hindlimb prints than female Wistar rats (M = 0.622; SE = 0.152), irrespective of time.
Hindlimb print width (cm)	Time*Strain	SD rats demonstrated significantly wider hindlimb prints at week 3 (*p* = 0.023), week 4 (*p* = 0.017), and week 6 (*p* = 0.025).
	Sex*Strain	On average, female SD rats (*M* = 1.160; *SE* = 0.146) showed 0.605 cm wider hindlimb prints than female Wistar rats (*M* = 0.555; *SE* = 0.146), irrespective of time.
Body speed (cm/s)	Time*Strain	On baseline testing, the body speeds of SD rats were 10.722 cm/s slower than Wistar rats, *p* = 0.021. However, on week 3, SD rats were 13.362 cm/s faster than Wistar rats, *p* = 0.023.
Duty cycle (%)	Time*Sex	On week 2, the hindlimb paws of female rats (*M* = 47.332; *SE* = 8.207) were placed on the ground 33.028% longer during the step cycle than male rats (*M* = 14.303; *SE* = 8.207), *p* = 0.022.

Note: interaction effects between variables are signified by an asterisk (*).

**Table 5:** Mean comparison between male and female in SD and Wistar strains following severe SCI.

Groups	Males (Mean, % Error)	Females (Mean, % Error)	Δ Mean (Male–Female) [95% CI]	*p*-Value
SD	88.10	84.21	3.89 [−9.80–17.57]	0.531
Wistar	79.48	77.55	1.93 [−11.76–15.61]	0.754
Δ Mean (SD–Wistar) [95% CI]	8.62 [−5.07–22.30]	−6.66 [−7.03–20.34]	---	---
*p*-value	0.184	0.294	*---*	*---*

**Table 6:** Significant interactions for additional CatWalk gait parameters assessed in male and female rats in both strains SD and Wistar following severe SCI in 7 weeks.

Parameter	Interaction/Effect	Key Observations
Step sequence/Regularity index (%)	Time*Sex	A significant interaction between time and sex was found, *F*(8, 64) = 0.086, *p* = 0.013. However, pairwise comparisons showed no significant differences between males and females at any particular timepoint.
Average max contact area (cm^2^)	Time*Sex	Significant two-way interactions were found between time and sex interaction, *F*(8, 64) = 5.232, *p* < 0.001, and also between time and strain, *F*(8, 64) = 11.448, *p* < 0.001. Females demonstrated significantly larger maximum hindlimb paw contact areas than males on baseline testing, *p* = 0.035. However, no significant differences were seen at any post-injury timepoint.
	Time*Strain	Wistar rats demonstrated significantly larger maximum hindlimb paw contact areas than SD rats on baseline testing, *p* = 0.006. However, no significant differences were seen at any post-injury timepoint. Also, significant differences were found between-subjects effect for sex, F (1, 8) = 5.760, *p* = 0.043, and strain, F (1, 8) = 11.116, *p* = 0.010.
	Sex	Irrespective of time or strain, female rats (M = 0.105; SE = 0.012) seemed to have an average of 0.039 cm^2^ larger maximum hindlimb paw contact areas than male rats (M = 0.066; SE = 0.012).
	Strain	Irrespective of time or sex, Wistar rats (M = 0.113; SE = 0.012) seemed to have an average of 0.055 cm2 larger maximum hindlimb paw contact areas than SD rats (M = 0.058; SE = 0.012).
Average step cycle (s)	Time*Strain	Significant two-way interaction was found between time and strain, *F*(8, 64) = 2.162, *p* = 0.042. SD rats demonstrated significantly longer average step cycles than Wistar rats on baseline testing, *p* = 0.030. However, no significant differences were seen at any post-injury timepoint.

Note: interaction effects between variables are signified by an asterisk (*).

## Data Availability

The original contributions presented in this study are included in the article/Supplementary Material. Further inquiries can be directed to the corresponding author.

## Supplementary Materials

The following supporting information can be downloaded at: https://www.mdpi.com/article/10.3390/brainsci15020191/s1, the Supplementary Materials Section contains all of the raw data for this study.

## Abbreviations

SCI	Spinal Cord Injury
SD	Sprague-Dawley
BBB	Basso–Beattie–Bresnahan
H&E	Hematoxylin and Eosin

## References

[1] Behrmann D.L., Bresnahan J.C., Beattie M.S., Shah B.R. (1992). Spinal cord injury produced by consistent mechanical displacement of the cord in rats: Behavioral and histologic analysis. J. Neurotrauma.

[2] Erschbamer M., Pernold K., Olson L. (2007). Inhibiting epidermal growth factor receptor improves structural, locomotor, sensory, and bladder recovery from experimental spinal cord injury. J. Neurosci..

[3] National Spinal Cord Injury Statistical Center (2024). Traumatic Spinal Cord Injury Demographics at a Glance, 2024 Birmingham.

[4] Wen X., Jiang W., Li X., Liu Q., Kang Y., Song B. (2024). Advancements in Spinal Cord Injury Repair: Insights from Dental-Derived Stem Cells. Biomedicines.

[5] Akhtar A.Z., Pippin J.J., Sandusky C.B. (2008). Animal models in spinal cord injury: A review. Rev. Neurosci..

[6] Bresnahan J.C., Beattie M.S., Stokes B.T., Conway K.M. (1991). Three-dimensional computer-assisted analysis of graded contusion lesions in the spinal cord of the rat. J. Neurotrauma.

[7] Nardone R., Florea C., Höller Y., Brigo F., Versace V., Lochner P., Golaszewski S., Trinka E. (2017). Rodent, large animal and non-human primate models of spinal cord injury. Zoology.

[8] Mills C.D., Hains B.C., Johnson K.M., Hulsebosch C.E. (2001). Strain and Model Differences in Behavioral Outcomes after Spinal Cord Injury in Rat. J. Neurotrauma.

[9] Wang F., Huang S.-L., He X.-J., Li X.-H. (2014). Determination of the ideal rat model for spinal cord injury by diffusion tensor imaging. NeuroReport.

[10] Ridlen R., McGrath K., Gorrie C.A. (2022). Animal models of compression spinal cord injury. J. Neurosci. Res..

[11] Wells J.E., Hurlbert R.J., Fehlings M.G., Yong V.W. (2003). Neuroprotection by minocycline facilitates significant recovery from spinal cord injury in mice. Brain.

[12] Rivlin A., Tator C. (1978). Effect of duration of acute spinal cord compression in a new acute cord injury model in the rat. Surg. Neurol..

[13] Cheriyan T., Ryan D.J., Weinreb J.H., Cheriyan J., Paul J.C., Lafage V., Kirsch T., Errico T.J. (2014). Spinal cord injury models: A review. Spinal Cord.

[14] von Euler M., Seiger Å., Sundström E. (1997). Clip compression injury in the spinal cord: A correlative study of neurological and morphological alterations. Exp. Neurol..

[15] Nistor-Cseppento C.D., Gherle A., Negrut N., Bungau S.G., Sabau A.M., Radu A.-F., Bungau A.F., Tit D.M., Uivaraseanu B., Ghitea T.C. (2022). The Outcomes of Robotic Rehabilitation Assisted Devices Following Spinal Cord Injury and the Prevention of Secondary Associated Complications. Medicina.

[16] Basso D.M., Beattie M.S., Bresnahan J.C. (1995). A sensitive and reliable locomotor rating scale for open field testing in rats. J. Neurotrauma.

[17] Basso D.M., Beattie M.S., Bresnahan J.C. (1996). Graded histological and locomotor outcomes after spinal cord contusion using the NYU weight-drop device versus transection. Exp. Neurol..

[18] Metz G.A., Whishaw I.Q. (2002). Cortical and subcortical lesions impair skilled walking in the ladder rung walking test: A new task to evaluate fore-and hindlimb stepping, placing, and co-ordination. J. Neurosci. Methods.

[19] Borrell J.A., Gattozzi D., Krizsan-Agbas D., Jaeschke M.W., Nudo R.J., Frost S.B. (2023). Chronic Stimulation Improves Motor Performance in an Ambulatory Rat Model of Spinal Cord Injury. J. Integr. Neurosci..

[20] Metz G.A., Whishaw I.Q. (2009). The ladder rung walking task: A scoring system and its practical application. J. Vis. Exp. JoVE.

[21] Timotius I.K., Bieler L., Couillard-Despres S., Sandner B., Garcia-Ovejero D., Labombarda F., Estrada V., Müller H.W., Winkler J., Klucken J. (2021). Combination of defined catwalk gait parameters for predictive locomotion recovery in experimental spinal cord injury rat models. Eneuro.

[22] Dickmann J., Gonzalez-Uarquin F., Reichel S., Pichl D., Radyushkin K., Baumgart J., Baumgart N. (2022). Clicker Training Mice for Improved Compliance in the Catwalk Test. Animals.

[23] Zheng G., Younsi A., Scherer M., Riemann L., Walter J., Skutella T., Unterberg A., Zweckberger K. (2021). The catwalk XT^®^ gait analysis is closely correlated with tissue damage after cervical spinal cord injury in rats. Appl. Sci..

[24] Llewellyn B. (2009). Nuclear staining with alum hematoxylin. Biotech. Histochem..

[25] Blight A.R. (1991). Morphometric analysis of a model of spinal cord injury in guinea pigs, with behavioral evidence of delayed secondary pathology. J. Neurol. Sci..

[26] Mestre H., Ramirez M., Garcia E., Martiñón S., Cruz Y., Campos M.G., Ibarra A. (2015). Lewis, Fischer 344, and Sprague-Dawley Rats Display Differences in Lipid Peroxidation, Motor Recovery, and Rubrospinal Tract Preservation after Spinal Cord Injury. Front. Neurol..

[27] Datto J.P., Bastidas J.C., Miller N.L., Shah A.K., Arheart K.L., Marcillo A.E., Dietrich W.D., Pearse D.D. (2015). Female Rats Demonstrate Improved Locomotor Recovery and Greater Preservation of White and Gray Matter after Traumatic Spinal Cord Injury Compared to Males. J. Neurotrauma.

[28] Zheng G., Zhang H., Tail M., Wang H., Walter J., Skutella T., Unterberg A., Zweckberger K., Younsi A. (2023). Assessment of hindlimb motor recovery after severe thoracic spinal cord injury in rats: Classification of CatWalk XT^®^ gait analysis parameters. Neural Regen. Res..

[29] Schmitt C., Miranpuri G.S., Dhodda V.K., Isaacson J., Vemuganti R., Resnick D.K. (2006). Changes in spinal cord injury–induced gene expression in rat are strain-dependent. Spine J..

[30] Koopmans G.C., Deumens R., Brook G., Gerver J., Honig W.M., Hamers F.P., Joosten E.A. (2007). Strain and locomotor speed affect over-ground locomotion in intact rats. Physiol. Behav..

[31] Swartz K.R., Fee D.B., Joy K.M., Roberts K.N., Sun S., Scheff N.N., Wilson M.E., Scheff S.W. (2007). Gender differences in spinal cord injury are not estrogen-dependent. J. Neurotrauma.

